# Effects on alcohol use of a Swedish school-based prevention program for early adolescents: a longitudinal study

**DOI:** 10.1186/s12889-016-3947-3

**Published:** 2017-01-03

**Authors:** Linda Beckman, Mikael Svensson, Susanna Geidne, Charli Eriksson

**Affiliations:** 1 Faculty of Medicine and Health, School of Health Sciences, Örebro University, 70182 Örebro, Sweden; 2 Department of Public Health, Karlstad University, Universitetsgatan 2, 65188 Karlstad, Sweden; 3 Health Metrics, The Sahlgrenska Academy, University of Gothenburg, Medicinaregatan 18G, 41390 Gothenburg, Sweden

**Keywords:** Adolescents, Evaluation, Intervention

## Abstract

**Background:**

The aim of the study was to address the lack of evaluations of school-based substance use prevention programs and to conduct a quasi-experimental evaluation of the alcohol use part of the Triad intervention.

**Methods:**

Eleven Swedish intervention schools (285 pupils) and three control schools (159 pupils) participated in the evaluation. Baseline measurements were conducted in 2011 before the alcohol part in the prevention program was implemented in the intervention schools (school year 6, ages 12–13). We estimated an Intention-To-Treat (ITT) Difference-in-Difference (DD) model to analyze the effectiveness of the intervention on subsequent alcohol use measured in grades 7, 8 and 9.

**Results:**

The main results show no effect on the likelihood of drinking alcohol or drinking to intoxication.

**Conclusions:**

The lack of positive effects highlights the need for policy-makers and public health officials need to carefully consider and evaluate prevention programs in order to ensure that they are worthwhile from school, health, and societal perspectives.

**Electronic supplementary material:**

The online version of this article (doi:10.1186/s12889-016-3947-3) contains supplementary material, which is available to authorized users.

## Background

Consumption of substances including tobacco, alcohol, and drugs is a significant health hazard for individuals and poses a challenge for public health program development [1–6]. A link between early adolescent onset of alcohol consumption and increased risk of developing dependence later in life has repeatedly been reported in retrospective cross-sectional studies [7] as well as in prospective longitudinal studies [8, 9]. Although alcohol dependence is partly explained by genetic factors, and environmental factors for alcohol initiation seem to be less of a risk factor [10], under-age alcohol consumption can interfere with school attendance, disrupt concentration, damage relationships, and potentially alter brain function or other aspects of development, health, and well-being [11, 12]. There are many factors that increase vulnerability to addiction, including developmental stage, exposure to early life adversity (ranging from abuse and neglect to and bullying), drug exposure, and genetic predisposition [13]. The impact on the developing brain is large when the child is younger; therefore, the interventions need to take developmental stage of the target group into consideration [14]. Moreover, there is evidence that knowledge acquisition, learning, and memory are vulnerable to alcohol use during adolescence [15]. In light of this, preventing adolescents from starting to use alcohol or other substances, or delaying the onset of use, is a fundamental task for public health work.

For decades, schools have been an important health-promoting setting [16, 17] and have been used as a platform for health promotion strategies and interventions [18] concerning, e.g., alcohol, drug, and tobacco consumption. However, although we seem to have good knowledge of which components work; due to the complex interactions of societal, social, and individual factors, interventions to prevent or reduce adolescents’ substance consumption are difficult to design, implement and evaluate [19, 20]. During recent decades, a range of preventive programs have been implemented internationally, as well as in Swedish schools. A Cochrane review [21] of the effectiveness of universal alcohol abuse prevention programs for children and adolescents found that less than half of the included studies (43.4%) reported significant beneficial results. In a recent systematic review by Agabio et al. [22] that included 12 randomized controlled trials not assessed in Foxcroft and Tsertsvadze’s study, 7 of these 12 additional reviewed interventions were more effective than control groups. However, a recent meta-analysis of reviews concluded that there was a small but consistent positive effect of school-based prevention programs; however, it is less clear what the “active ingredient” of such success is [23].

Most evidence-based prevention programs are evaluated in the US in small and highly controlled trials with high levels of implementation fidelity [24], and the results are therefore not easily transferred to other contexts or to broader program implementations in general. In Sweden, few alcohol prevention programs have been evaluated, so there is a general lack of evidence on the effectiveness of such programs [25, 26]. Moreover, there are important challenges in context adaptation of foreign programs into other national settings [27, 28]. Therefore, the aim of the study was to address the lack of evaluations of school-based substance use prevention programs and to conduct a quasi-experimental evaluation of the alcohol use part of the Triad.

### Alcohol consumption

Internationally, mean alcohol consumption among adolescents varies substantially among countries. The ESPAD study [29] found that among 16-year-old adolescents, girls’ alcohol consumption ranged from 21 centiliters of 100% alcohol per year in Romania to 137 centiliters on the Isle of Man. For boys it ranged from 71 centiliters per year in Ukraine to 173 centiliters on the Isle of Man. Boys generally report higher annual alcohol consumption than girls in all countries except Iceland and Norway. When “heavy episodic drinkers” (five or more drinks in a row during the last 30 days) are considered, the frequency for girls varied from 1.5 times in the last 30 days in Romania to 3.7 times on the Isle of Man. The corresponding figures for boys were 2.2 (Iceland, Romania and Switzerland) and 3.7 (Estonia and Malta) times in the last 30 days. Overall, boys reported a significantly higher frequency of heavy of drinking than did girls in all countries except Sweden and Iceland.

Swedish law, as in many countries, requires a person to be 18 years old to purchase alcoholic beverages containing more than 2.25% alcohol by volume. At the state-monopoly alcohol stores, the age limit for purchasing alcoholic beverages containing over 3.5% alcohol by volume is 20 years [30]. The national trend in Sweden is toward decreasing alcohol consumption among adolescents, however this could be explained by a polarization of drinking [31, 32]. Currently, data show that 47% of 15-year-olds (43% of boys and 50% of girls) in Sweden consume alcohol [33].

### Preventive interventions

Substance use prevention programs in schools were initially characterized by short term interventions and fear arousal, lecturing students about the dangers and long-term health consequences of substance consumption [34, 35]. Such interventions were rarely successful and generally lacked a theoretical foundation that considered developmental factors, social influences, and other potential contributors to adolescent substance consumption. Today, we know more about which components are successful in interventions, but few of the many extant programs include these features or evaluate their effects with well-designed research [36]. From a learning perspective it has been argued that interactive strategies, combining practice in social skills and educational knowledge transmission, increase the likelihood of success in a prevention program [24, 25, 37, 38]. Interventions that target more than one risk factor also seem to be more effective [34, 39]. Methods for strengthening social, emotional, behavioral, cognitive, and moral competencies; building self-efficacy; improving social relations with adults, peers, and younger children; providing structure and consistency in program delivery; and intervening with youth for a longer period of time are further effective components [40–42]. There is no evidence that resistance skills training has an effect on reducing substance use [36]. There is also no evidence that more intensive programs are more effective than less intensive ones [36]. Reviews have also tried to elucidate the effectiveness of school, family, and multicomponent programs, with some ambiguous results. While a Cochrane review [21] reported little evidence of the effectiveness of multicomponent interventions, compared with single-component interventions, in reducing alcohol misuse among youths, e.g., Catalano et al. [40]. Another program, originally from Finland but used in many European countries, targets pupils directly in class and uses competition as motivation. This is known as the “Smoke-free class competition” (SFC). The SFC targets only tobacco use and has been evaluated positively in a meta-analysis [43]. The core of the SFC is that entire school classes compete by signing a contract in which they promise to be smoke-free for the next 6 months. If they succeed they can win prizes in a lottery. The methodological approach of using prizes as rewards relies on frameworks such as learning theory [44] and social learning theory [45], where the behavior of remaining smoke-free becomes attractive and worthwhile; also, classmates are important to adolescents and function as influential role models for smoking initiation. Incentives, such as prizes or rewards, may be effective in improving single health behaviors, as shown by J Kavanagh, A Oakley, A Harden, A Trouton and C Powell [46]. However, this has not been the case for complex health behaviors.

Although Swedish schools have adopted a range of different prevention programs, published evaluations on programs targeting the pupils directly, in class, are scarce. Programs shown to be effective internationally lack counterparts in Sweden [47, 48]. One exception is the “Unplugged” program, which aims to postpone substance use and reduce future use. The program was implemented in 14 Swedish schools and in another set of schools in seven European countries. The program employs a high degree of interactive pedagogy, e.g., by involving pupils as leaders, providing education in life skills, holding parent meetings, and training social skills and norms related to alcohol and narcotics [47, 49]. On a European level, the results showed that the program was more effective than traditional substance use education. However, in Sweden there were no significant differences between control and treatment schools. It was argued that the program’s lack of measurable effect may be due to the fact that many of the ordinary prevention strategies implemented in control schools offer a high degree of interactivity similar to that in the Unplugged program [49].

The Triad (in Swedish “*Triaden*”) is an ongoing intervention administered by the non-governmental organization (NGO) Team 49. An entire school can purchase the program, but implementation occurs at the classroom level. Eleven intervention schools and three control schools participated. The authors of this study did not participate in the development of the program. Our aim with this study was to address the lack of evaluations of school-based substance use prevention programs and to conduct a quasi-experimental evaluation of the alcohol use part of the Triad intervention. The reason why we only address alcohol use were that smoking, and other drugs are very uncommon in these ages hence there would be too few pupils included in the analysis.

The Triad consists partly of components that have been shown to be successful but also includes some weaknesses (described under Structure and Components of the Program). Our investigation will add to the body of evidence related to school-based intervention programs. To our knowledge, this is the first study to evaluate a Swedish program targeting this age group during school hours. This study focuses on one of the three components of the Triad program – preventing substance use (“Fighting Drugs”) – by following pupils from school year 6 (baseline) to school year 9.

## Methods

### Structure and components of the intervention

The intervention the Triad is administered by the non-governmental organization (NGO) Team 49. The NGO has no religious or temperance-related affiliation. The purpose of the Triad is to increase pupils’ resistance skills through understanding and awareness of the harmful consequences of drugs and delinquent behavior, which in turn is believed to influence them to refrain from drugs and crime. The entire class participates and constitutes a team in arranged competitions with prizes. The program is built on different themes for each grade: Grade 4 (10- and 11-year-olds) includes traffic education; grade 5 (11- and 12-year-olds) aims to strengthen pupils’ morality and ethics and to prevent non-normative behavior such as delinquency, bullying, and shoplifting; and grade 6 (12- and 13-year-olds) encourages pupils to refrain from using alcohol, tobacco, and other drugs. The project started in 2011 with these three grade cohorts. This particular study is based on the grade 6 cohort, which only received the substance use theme of the prevention program in grade 6, and a follow-up at the beginning of grades 7, 8 and 9.

The information and educational materials are available to the teachers on the Triad website (via a personal login). A brochure can be downloaded with information about such things as how to use the materials, news, teacher tutoring (including pedagogical tips for planning the lesson), and fact sheets. Support is available if needed, and the teacher decides how to schedule the tasks and how much time each task will take.

The teachers are provided with suggested tasks for the class that aim to increase awareness in a certain area (e.g., drugs). The tasks align with the content and timeframe of the school curriculum (4–5 tasks, estimated time 80 min/task). An example of a task connected to the subject of math is: “Calculate, based on the knowledge that alcohol cost the society 5 billons Euro, how much money each individual would get out of this sum. It lives 9 billion inhabitants in Sweden”. A further example, connected to art, is “Draw a transparent person and show where in the body different diseases can strike us if we begin to drink. Present the results in front of the class.” In addition, about four 10-min informational films/grade about the current topic shows the consequences and hazards of, e.g., using drugs. Powerpoint slideshows with discussion material are also available. The Triad program also seeks to involve parents by allowing the students do tasks at home (decided by the teacher), e.g., discussing or investigating a certain topic together with the family: “Ask a grownup in your home if they know why people drink. Ask them to tell you about a situation where some grownups they met have behaved badly while drinking. Write down their story and tell the story to the class.” Or, “Ask a grownup in your home if they know why other grownups buy alcohol for underage adolescents despite knowing about the hazards of drinking alcohol.” When a task is completed, the teacher reports this via the website, and also evaluates each task.

The incentive for participating and completing the tasks is a lottery where the whole class can win prizes at the end of the school year; all tasks must be completed in order to participate. The program includes three competitions, one for each grade, and ends with a grand finale together with the parents, where the prizes are diplomas and grants dedicated for a particular experience. The finale is arranged in cooperation with the municipality and/or local businesses.

This program includes some components that have been shown to be effective in other preventive substance use programs, and some that have not. The main components, shown in Table 1, include (1) increasing resistance skills through understanding and awareness of the harmful consequences of drugs and delinquency, (2) involving parents, and (3) a lottery contest where the whole class can win prizes, which seems to be an incentive for participants to complete the tasks.

**Table 1:** Components of the Triad program

• Increasing resilience skills through understanding and awareness about consequences and hazards of e.g., using drugs by showing ~ 4 10-min informational films per grade about the relevant topic, and showing and/or using power points with discussion material.
• Involving parents.
• The incentive for participating and completing the tasks is a lottery where the entire class can win prizes at the end of the school year.

### Participants

Eleven intervention schools (285 intervention pupils, participation rate 93%) and three control schools (159 control pupils, participation rate 92%) participated at baseline in 2011 (school year 6). When pupils in the intervention schools left grade 6 and went to grade 7 (junior high school) they attended the same junior high school. At baseline, participation was somewhat higher in the control schools than in the intervention schools (91 and 85% respectively; Table 1). Attrition was also higher in the intervention schools. Table 2 shows demographic data for the participants. The intervention and control schools were similar with regard to gender and ethnic background, but pupils in the intervention schools had better housing conditions and greater cultural capital, and more often lived in a nuclear family.

**Table 2:** Participants in the intervention and control schools at baseline and at follow up

	Autumn 2011 school year 6 (baseline)	Autumn 2012 school year 7	Autumn 2013 school year 8	Autumn 2014 school year 9
Intervention schools	Girls: *n* = 141	Girls: *n* = 119	Girls: *n* = 128	Girls: *n* = 120
	Boys: *n* = 143	Boys: *n* = 112	Boys: *n* = 111	Boys: *n* = 111
	Total: 284/334	Total: 231/334	Total: 239/334	Total: 231/334
	(85.0%)	(69.2%)	(71.6%)	(69.2%)
Control schools	Girls: *n* = 72	Girls: *n* = 68	Girls: *n* = 68	Girls: *n* = 63
	Boys: *n* = 73	Boys: *n* = 68	Boys: *n* = 66	Boys: *n* = 58
	Total: 145/160	Total: 136/160	Total: 134/160	Total: 121/160
	(90.6%)	(85%)	(83.8%)	(75.6%)

### Procedure

Swedish municipalities, which are responsible for both funding and arranging primary and secondary education, were informed about the study and invited to participate. The municipalities decided which schools would participate, and then informed the schools about the program. Hence, schools did not self-select into the intervention; therefore, the principal and teachers could be positive, neutral or negative toward the program. This could arguably improve external validity of the design relative to that of a typical randomized controlled trial in terms of predicting the effectiveness of the program if it were to be rolled out on a larger scale. All participating schools signed a contract whereby intervention schools undertook to conduct the Triad program in its entirety for three years, and control schools promised to continue with their usual preventive work but not to implement any specific program.

The pupils’ parents received information about the study’s aim, the voluntary nature of their compliance, and their right to withdraw from the study at any time. Parents who did not want their children to participate were asked to contact the research team. Participants were asked during school hours to fill in a questionnaire about family background, tobacco, alcohol, and self-rated health. The data collection was implemented by the research team.

#### Ethics

The research conformed to the Helsinki Declaration and ethical approval was obtained from the Regional Ethics Review Board in Uppsala (reg. no 2011/213).

### Empirical strategy

This study is part of a larger study focusing on “School as a setting for alcohol and drug prevention” in a special venture financed by the Swedish government [50, 51]. The program was designed and implemented by the NGO, and the research team was responsible for the research and evaluation activities.

### Measurements

#### Outcome variables

Alcohol consumption was measured with two questions. The first was “Have you ever drunk alcohol?” The response alternatives were: (1) “No, I have never drunk alcohol”; (2) “I have taken a sip from someone else’s glass”; (3) “I have drunk alcohol on one occasion”; and (4) “I have drunk alcohol on more than one occasion.” The alternatives were dichotomized into “No, I have not drunk alcohol” (1 and 2) and “Yes, I have drunk alcohol” (3 and 4). The second question was “Have you ever been intoxicated by alcohol?” and had the following response alternatives: (1) “No, I have never been intoxicated”; (2) “No, I have tasted it but I have never been intoxicated”; (3) “Yes, once”; (4) “Yes, 2–3 times”; (5) “Yes, 4 times or more”; (6) “Yes, every time I used alcohol.” We dichotomized this item such that response options 1 and 2 were defined as “No, I have never been intoxicated” and options 3–6 as “Yes, I have been intoxicated.”

#### Control variables

Socioeconomic status included the immigrant status of the pupil, mother, and father separately (“born in Sweden,” yes/no); family structure (“nuclear family,” yes/no); living situation (“living with one parent” “shared residence,”); type of residence “if living in rental apartment (or otherwise house, owned apartment)”; number of books at home trichotomized into “many books” (if >250 books in household), “medium books” (if 50–250 books), “few books” (if <50 books). Other control variables included gender (boy/girl) and school year (6, 7, 8, 9) (see Table 2).

### Regression analysis

The intervention is conducted at the school level, and the adolescents could not influence whether they participated in the intervention or control group. Thus, we estimated a standard Intention-To-Treat (ITT) Difference-in-Difference (DD) logit model (given that the outcome variables are binary) as described in equation (1), where *Y*_*it*_ represent the different outcome variables *Alcohol Consumption* and *Intoxication* for adolescent *i* in grade *t*:

1 $$ \Pr \left({Y}_{it}=1\right)=\alpha +{\beta}_0 Interventio{n}_i+{\beta}_1Pos{t}_t+{\beta}_2 Interventio{n}_{it}\times Pos{t}_t+\theta {\boldsymbol{X}}_{\boldsymbol{it}}+{\varepsilon}_{it}. $$

*Intervention* is a dummy variable indicating whether an adolescent attends an intervention school (equal to 1 if so, and 0 otherwise), and *Post* is a dummy variable for post-treatment data (i.e., equal to 1 for grade 7, 8 or 9, and equal to 0 for grade 6). The interaction term *Intervention × Post* is the causal effect of the program on the outcome variables given our assumed identification strategy, and thus *β*_2_ is the coefficient of main interest. Finally, ***X***_***it***_ is a vector of individual-specific control variables and *ε*_*it*_ is the error term. Considering that we had repeated measures at the individual level (panel/longitudinal data), we clustered the standard errors at the individual level. Failure to do so would bias our standard errors and thus the statistical significance.

The main standard identifying assumption for drawing causal conclusions in our model, as in the typical DD setup, is that intervention and control schools would follow the same trend in alcohol consumption and intoxication albeit from different starting points (the common-trend assumption). One source of concern is that we know that the adolescents in the intervention and control schools differ slightly on a number of observable characteristics (Table 3). For example, there is a higher proportion of adolescents in nuclear families in the intervention schools. If such adolescents have a significantly different trend in their alcohol consumption behavior over time, this may bias our results. Note however that such differences affecting levels of alcohol intake are not a problem, given that our identification relies on differences in the trend (not levels). Still, in order to examine the possible influence of such confounders on differential trends in alcohol consumption, we introduced a number of control variables (***X***_***it***_) in the regressions. As a robustness check we also used propensity score matching techniques to create a balance between treatment and control schools in observable characteristics and ran the analysis on the balanced sample. We also conducted multilevel regressions, directly taking the hierarchical nature of the data into account, which yielded the same qualitative interpretations and results. 1

**Table 3:** Descriptive statistics: mean of control variables

		Intervention schools	Control schools
		(*n* = 172)	(*n* = 101)
Boy	=1 if pupil is a boy	0.47	0.50
Rental	=1 if pupils lives in rental apartment, and 0 if in owned house or apartment	0.17***	0.29
Swedish	=1 if pupil was born in Sweden with at least 1 Swedish parent	0.96**	0.92
Nuclear	=1 if pupil lives with both biological parents	0.76	0.73
Shared	=1 if lives every other week with mother/father	0.13**	0.07
Single parent	=1 if only lives with one parent	0.11**	0.16
Books many	=1 if >250 books in household	0.20**	0.14
Books medium	=1 if 50–250 books in household	0.53***	0.43
Books few	=1 if <50 books in household	0.27***	0.43

Note: **p = 0.01; ***p <0.001, statistically significant difference compared to control schools (two-sided *t*-test)

## Results

**Fig. 1:**
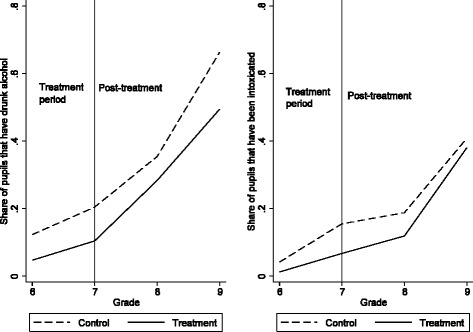
The trend in proportion of students using alcohol and drinking to intoxication

We begin by graphically depicting the trends in *Alcohol Consumption* and *Intoxication* in the intervention and control schools from grade 6 (baseline) to grades 7, 8, and 9. Figure 1 shows a similar increase in *Alcohol Consumption* for the intervention schools as for the control schools. For *Intoxication* the picture is less clear, with a less steep increase in the intervention schools from grades 6 to 7, but a steeper increase for grade 8 and grade 9. The proportion of students using alcohol increases as expected from a starting point in grade 6 at 0.05–0.12 to 0.50–0.66 in grade 9, whereas the numbers for intoxication are 0.01–0.04 in Grade 6 to 0.38–0.40 in grade 9.

The results for both *Alcohol Consumption* and *Intoxication* show no statistically significant effect of the program (with or without controls). We therefore cannot reject the null hypothesis that the intervention has no effect on the likelihood of adolescents using alcohol or drinking themselves to intoxication at least once.

Table 4 also reveals that the variable *Post* is positive and statistically significant in all models, which simply represents the fact that alcohol consumption and intoxication increase in higher grades in both the intervention and control schools. We report marginal effects, with coefficient estimates implying that alcohol consumption is 22–23 (intoxication 13–15) percentage points more likely in grades 7–9 than in the baseline year (grade 6). The variable *Intervention* is negative and is significant for alcohol consumption, which should be interpreted as meaning that pupils in the intervention schools are 20–22 percentage points less likely to use alcohol (a “level” effect), but that this effect was already present at baseline (before the intervention), a result also observable in Fig. 1. As for the individual-level control variables, very few are statistically significant predictors of alcohol consumption or intoxication.

**Table 4:** Main results of the effect of The Triad on alcohol consumption and intoxication: results shown as marginal effects (standard errors)

	Alcohol consumption		Intoxication	
	Model (1)	Model (2)	Model (3)	Model (4)
Intervention	−0.20**	−0.22**	−0.15	−0.24
	(0.094)	(0.096)	(0.114)	(0.148)
Post	0.23***	0.22***	0.15***	0.13***
	(0.033)	(0.033)	(0.030)	(0.030)
Intervention × Post	0.10	0.12	0.10	0.16
	(0.084)	(0.086)	(0.089)	(0.107)
Boy		0.01		−0.00
		(0.034)		(0.020)
Rental		−0.04		−0.03
		(0.045)		(0.026)
Swedish		−0.05		0.02
		(0.067)		(0.037)
Nuclear		−0.03		0.01
		(0.055)		(0.034)
Shared		0.03		0.03
		(0.076)		(0.054)
Books many		−0.04		−0.01
		(0.044)		(0.028)
Books medium		−0.07*		−0.02
		(0.037)		(0.023)
Observations	1062	1040	1062	1039

Note: *p = 0.05; **p = 0.01; ***p <0.001, statistically significant difference compared to control schools (two-sided *t*-test)

Table 4 used information from all years. To examine the robustness of the results, we also conducted analyses in which we compared the baseline alcohol consumption and intoxication results to those in each of the other grades. These data appear in Additional file 1: Table S5 (grade 7 comparison), Additional file 1: Table S6 (grade 8 comparison) and Additional file 1: Table S7 (grade 9 comparison) (included as Additional file).

The results from Table 4 persist in these analyses as well. Hence we cannot reject the null hypothesis of no effect in any of the estimations in Additional file 1: Tables S5-S7.

## Discussion

The aim of the study was to conduct a quasi-experimental evaluation of the alcohol use part of the Triad intervention. Our main results showed no effect on the likelihood of alcohol consumption or intoxication. These results are in line with previous literature showing that many school-based interventions do not have the intended effect [21].

We know that a successful program should be based on and tested in well-designed research programs [36]. It should include interactive strategies [24, 25, 37, 38], target more than one risk factor [34, 39], strengthen social, emotional, behavioral, cognitive, and moral competencies; and provide structure and consistency in program delivery [40–42]. The premise for the Triad program is not informed by research, e.g., into the ways that developmental factors and social influences contribute to adolescent substance use [34, 39]. Although the program does not rely solely on information transmission, the materials do include awareness-raising materials about the hazards of drinking, which can be seen as “fear arousal,” a component found not to be successful in interventions [34, 35]. The program does include some components that are corroborated by research, such as interactive learning strategies [24, 25, 37, 38], but is partly based on resistance training, which is not supported in the literature [36]. Encouragement is fueled by rewards through competitions and prizes, which have received some support for effectiveness in improving health behaviors [46]. Further, the Triad intervention followed the pupils for an extended period of time and focused on strengthening social and moral competencies and improving social relations with adults, which is supported by research [40, 41].

There are many possible reasons that we failed to detect positive effects of the program, besides weaknesses or ineffectiveness of program components. One is that the participants in our dataset only received the “Fighting Drugs” intervention theme in grade 6; they did not receive the interventions associated with other behavioral themes in grades 4 and 5. It may be that receiving all themes is more effective, as the complete program targets several risk behaviors [34, 39]. However, our participants did receive the intervention that was directly related to alcohol and substance use, which limits the concern that they were not exposed to the full program.

A second possible reason for the observed lack of benefit of the program is that it was poorly implemented. Although no data were available on program fidelity in the schools, the fact that implementation was conducted by an NGO, rather than left to the various school personnel, probably improved fidelity.

The fact that participating schools were not self-selected, but chosen by the municipality, may have reduced the involvement of the teachers in program implementation and effectiveness. But for evaluation and policy purposes, this is less interesting, because if the program were to be scaled up based on a decision by policy makers, and moved outside a more controlled experiment, such a real-world policy context would be more closely mimicked by our evaluation design; i.e., the emphasis is on “effectiveness rather than efficacy.”

Another potential reason for the absence of a beneficial effect, similar to discussions by A-M Lindahl and M Rosaria Galanti [49], is that control schools are likely to work with other prevention strategies that are similar to Triad. However, if schools in general are already applying some relevant prevention strategies, this reduces the need for more formal programs like Triad.

Further, it could be that norms and peer-pressure may offer stronger incentives than does the program itself [52]. The program did not have a specific component addressing the development of socio-emotional skills including role-play activities, which have been a core component in many of the programs that have proven effective when implemented as intended. In addition, the primary prevention approach that Triad offers may be less likely to impact the adolescent behavior in question. In fact, studies have shown that secondary prevention programs for at-risk or high-risk youth for whichever behavior the adolescent is at risk for, may well be more successful [53, 54].

A limitation in our empirical analysis is that, as is typical in quasi-experiments, the identification of a causal effect of the program is based on a common-trend assumption, i.e., that the intervention and control schools would have had the same trend in alcohol consumption without the intervention. The common-trend assumption is impossible to test, because we cannot observe the trend in the intervention schools without the intervention. The adolescents in the intervention and control schools differed slightly on a number of observable characteristics, and if these affected the trend in their alcohol consumption behavior over time, it could have biased our results. However, we took a number of steps to try to address the potential problems that arise in the quasi-experimental context. First, we introduced a number of control variables in the regressions (which is not necessary if there is random assignment), which can increase the efficiency of the analysis. Also, we conducted a number of robustness checks with other empirical approaches, including propensity score matching as well as multilevel regressions. Our results are very robust with regard to the different empirical strategies. Another limitation was that we only analyzed alcohol use since the numbers of pupils smoking or using other drugs are very rare in these ages.

## Conclusions

We found a non-significant impact on alcohol consumption and intoxication of the “Fighting Drugs” component of the Triad intervention for early adolescents. If these results are further strengthened by similar results from evaluations of the two other themes of the program, the program should be re-developed or removed from schools’ health promotion strategies. The lack of positive effects thus indicates that the program makes poor use of resources and time. It also raises concerns regarding other prevention programs that rely to a large extent on similar components, and it calls for more systematic and formal evaluations of health interventions taking place in the school setting.

## Additional file

Additional file 1:
**Table S5.** Comparing grade 7 alcohol consumption and intoxication with that of grade 6: results shown as marginal effects (standard errors). **Table S6.** Comparing grade 8 alcohol consumption and intoxication with that of grade 6, results shown as marginal effects (standard errors). **Table S7.** Consumption, results shown as marginal effects (standard errors). (DOC 84 kb)

## Abbreviation

NGO: Non-government organizations

## Footnotes

1 We have performed these analyses and they give the same conclusions. Results will be supplied on request.
