# Supplementary material for: Effects on alcohol use of a Swedish school-based prevention program for early adolescents: a longitudinal study
Source: BMC Public Health. 2017 Jan 3;17:2. doi: 10.1186/s12889-016-3947-3 (PMC5209812; doi:10.1186/s12889-016-3947-3)
Supplement: Additional file 1: — Table S5. Comparing grade 7 alcohol consumption and intoxication with that of grade 6: results shown as marginal effects (standard errors). Table S6. Comparing grade 8 alcohol consumption and intoxication with that of grade 6, results shown as marginal effects (standard errors). Table S7. Consumption, results shown as marginal effects (standard errors). (DOC 84 kb) [file 12889_2016_3947_MOESM1_ESM.doc]

**Additional file 1: TABLE S5-S7**

**Table S5** Comparing grade 7 alcohol consumption and intoxication with that of grade 6: results shown as marginal effects (standard errors)

|  | Alcohol consumption | | Intoxication | |
| --- | --- | --- | --- | --- |
|  | Model (1) | Model (2) | Model (3) | Model (4) |
| Intervention | -0.10** | -0.11** | -0.05 | -0.08* |
|  | (0.051) | (0.050) | (0.037) | (0.044) |
| Post | 0.05* | 0.05* | 0.03* | 0.02 |
|  | (0.027) | (0.026) | (0.019) | (0.016) |
| Intervention × Post | 0.02 | 0.03 | 0.01 | 0.02 |
|  | (0.045) | (0.045) | (0.029) | (0.032) |
| Boy |  | 0.05* |  | 0.01 |
|  |  | (0.028) |  | (0.015) |
| Rental |  | -0.02 |  | -0.01 |
|  |  | (0.035) |  | (0.014) |
| Swedish |  | 0.01 |  |  |
|  |  | (0.057) |  |  |
| Nuclear |  | 0.02 |  | 0.01 |
|  |  | (0.043) |  | (0.017) |
| Shared |  | 0.05 |  | 0.02 |
|  |  | (0.084) |  | (0.047) |
| Books many |  | -0.03 |  | 0.01 |
|  |  | (0.033) |  | (0.017) |
| Books medium |  | -0.03 |  | -0.01 |
|  |  | (0.028) |  | (0.016) |
| Observations | 532 | 521 | 528 | 488 |

Robust standard errors in parentheses

*** p<0.01, ** p<0.05, * p<0.1

**Table S6** Comparing grade 8 alcohol consumption and intoxication with that of grade 6, results shown as marginal effects (standard errors)

|  | Alcohol consumption | | Intoxication | |
| --- | --- | --- | --- | --- |
|  | Model (1) | Model (2) | Model (3) | Model (4) |
| Intervention | -0.15** | -0.17** | -0.08 | -0.11 |
|  | (0.071) | (0.072) | (0.059) | (0.071) |
| Post | 0.18*** | 0.17*** | 0.09*** | 0.07** |
|  | (0.043) | (0.042) | (0.034) | (0.031) |
| Intervention × Post | 0.10 | 0.11 | 0.04 | 0.07 |
|  | (0.073) | (0.076) | (0.055) | (0.065) |
| Boy |  | 0.04 |  | 0.01 |
|  |  | (0.032) |  | (0.015) |
| Rental |  | -0.03 |  | -0.02 |
|  |  | (0.039) |  | (0.017) |
| Swedish |  | -0.03 |  | -0.02 |
|  |  | (0.074) |  | (0.037) |
| Nuclear |  | -0.02 |  | -0.01 |
|  |  | (0.055) |  | (0.023) |
| Shared |  | 0.04 |  | 0.02 |
|  |  | (0.076) |  | (0.037) |
| Books many |  | -0.01 |  | -0.01 |
|  |  | (0.046) |  | (0.019) |
| Books medium |  | -0.02 |  | -0.00 |
|  |  | (0.035) |  | (0.016) |
| Observations | 532 | 522 | 531 | 520 |

Robust standard errors in parentheses

*** p<0.01, ** p<0.05, * p<0.1

**Table S7** Consumption, results shown as marginal effects (standard errors)

|  | Alcohol consumption | | Intoxication | |
| --- | --- | --- | --- | --- |
|  | Model (1) | Model (2) | Model (3) | Model (4) |
| Intervention | -0.20** | -0.20** | -0.14 | -0.20* |
|  | (0.091) | (0.092) | (0.099) | (0.114) |
| Post | 0.46*** | 0.45*** | 0.28*** | 0.24*** |
|  | (0.056) | (0.057) | (0.067) | (0.071) |
| Intervention × Post | 0.06 | 0.08 | 0.13 | 0.18 |
|  | (0.097) | (0.099) | (0.104) | (0.124) |
| Boy |  | 0.01 |  | -0.01 |
|  |  | (0.040) |  | (0.018) |
| Rental |  | -0.03 |  | -0.03 |
|  |  | (0.052) |  | (0.024) |
| Swedish |  | -0.07 |  | 0.04 |
|  |  | (0.092) |  | (0.030) |
| Nuclear |  | -0.04 |  | 0.02 |
|  |  | (0.066) |  | (0.027) |
| Shared |  | -0.01 |  | 0.05 |
|  |  | (0.084) |  | (0.065) |
| Books many |  | -0.05 |  | -0.02 |
|  |  | (0.052) |  | (0.025) |
| Books medium |  | -0.11** |  | -0.03 |
|  |  | (0.045) |  | (0.025) |
| Observations | 538 | 527 | 535 | 524 |

Robust standard errors in parentheses

*** p<0.01, ** p<0.05, * p<0.1
